# Methodological Quality and Content of Guidelines on Early Childhood Allergy Prevention: A Systematic Assessment and Content Analysis

**DOI:** 10.1111/mcn.13779

**Published:** 2024-12-13

**Authors:** Katharina Sieferle, Eva M. Bitzer

**Affiliations:** ^1^ Public Health & Health Education University of Education Freiburg Freiburg im Breisgau Germany

**Keywords:** asthma, atopic, clinical practice guideline, dermatitis, dietary guidelines, food allergy, hypersensitivity, immediate, primary prevention

## Abstract

Recommendations on Early Childhood Allergy Prevention (ECAP) are found in Clinical Practice Guidelines (CPG) and Food‐Based Dietary Guidelines (FBDG). This synthesis of guidelines aims to compare the methodological quality and content of recommendations in CPGs and FBDGs for ECAP. We searched MEDLINE, the FAO directory of FBDGs and other guideline databases, including the Association of the Scientific Medical Societies in Germany (AWMF), the WHO and the Guideline International Networks database on clinical guidelines (GIN) for CPGs and FBDGs about ECAP and child nutrition. Guidelines had to be published from 2010 onwards, target infants or pregnant/breastfeeding women and contain recommendations on primary preventative interventions to decrease the onset of IgE‐mediated allergies, including atopic eczema or asthma. We retrieved a sample of 36 guidelines (23 CPGs, 13 FBDGs) and assessed their methodological quality with the Appraisal of Guidelines for Research and Evaluation tool (AGREE) II. On a subset of recommendations, we performed an in‐depth analysis by the type of intervention for direction and strength of recommendation and level of evidence. Descriptive analysis was conducted with SPSS 27. CPGs score higher than FBDGs in most AGREE domains (3, 4, 5 and 6). The 36 guidelines contain 287 recommendations on ECAP, with 70 addressing the introduction of complementary foods and common allergens. We found only slight differences between those recommendations in CPGs and FBDGs. FBDGs on ECAP are of lower quality than CPGs. This does not affect their recommendations on the introduction of complementary foods and common allergens but may compromise their trustworthiness.

## Summary

- The methodological quality of guidelines on Early Childhood Allergy Prevention (ECAP), particularly Food‐Based Dietary Guidelines, is low, with deficiencies especially in the rigour of development, applicability, and editorial independence of the guidelines.
- Most guidelines on ECAP make recommendations on the introduction of complementary foods and recommend the introduction between 4 and 6 months, with little variation.
- Recommendations on the introduction of common allergens show comparably more variation.
- The slight differences in these recommendations may arise from the variations in methodological quality but also the time of publication of the guidelines, reflecting the evidence available at that time.

## Graphical abstract

Clinical Practice Guidelines and Food‐Based Dietary Guidelines differ in methodological quality, but their recommendations on Early Childhood Allergy Prevention are fairly consistent. However, low transparency in guideline development can still lead to reduced trust in the guideline, low implementation and insufficient adherence to the recommendations.

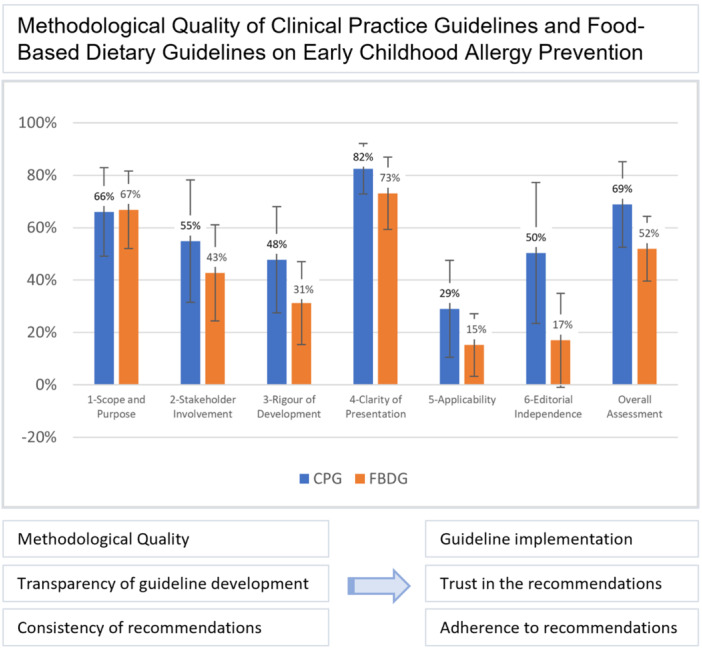

## 1 Introduction

The prevalence of childhood allergies is high, with approximately 20% of the global population being affected by allergic rhinitis, eczema, asthma or food allergies (Dierick et al. 2020; Pawankar 2014; Bantz, Zhu, and Zheng 2014). These atopic diseases are associated with a reduced quality of life and a high economic burden, and the high prevalence makes their prevention an important public health concern (Pawankar 2014; Pawankar et al. 2013).

Recommendations on Early Childhood Allergy Prevention (ECAP) are found in Clinical Practice Guidelines (CPG) and Food‐Based Dietary Guidelines (FBDG). The US Institute of Medicine defines CPGs as ‘statements that include recommendations intended to optimise patient care that are informed by a systematic review of evidence and an assessment of the benefits and harms of alternative care options’ (Institute of Medicine US Committee on Standards for Developing Trustworthy Clinical Practice Guidelines et al. 2011, p. 4). CPGs are primarily targeted towards healthcare professionals to aid in the decision‐making process for an appropriate patient care (Arbeitsgemeinschaft der Wissenschaftlichen Medizinischen Fachgesellschaften AWMF—Ständige Kommission Leitlinien 2012) but might also be used by affected patients and in policy processes (Eccles et al. 2012). FBDGs, on the other hand, primarily target the general population (Bechthold et al. 2018). According to the World Health Organisation (WHO), the Food and Agriculture Organisation (FAO) and the European Food Safety Authority (EFSA), FBDGs are science‐based policy recommendations for healthy eating. Since FBDGs are primarily targeted at consumers, they should be practical, consistent and easily understandable. They should be based on scientific evidence, current dietary practices and prevailing public health problems, not only on nutrient requirements (Bechthold et al. 2018; World Health Organisation 1998). Especially in fields with rapidly evolving evidence like ECAP, CPGs and FBDGs are important to provide practitioners in ECAP and child nutrition (CN) with reliable guidance. FBDGs and CPGs have the potential to improve healthcare quality and safety by translating research into practice (Institute of Medicine US Committee on Standards for Developing Trustworthy Clinical Practice Guidelines et al. 2011; Qaseem 2012). However, considerable concern has been expressed by physicians, consumer groups and other stakeholders about the quality of the processes supporting the development of FBDGs and CPGs (Bindslev et al. 2013; Montagnese et al. 2015). Among the factors undermining the quality and trustworthiness are limitations in systematic reviews upon which CPGs are based, lack of transparency of development groups' methodologies (particularly regarding evidence quality and strength of recommendation appraisals) and unmanaged conflicts of interest (COI) (Hansen et al. 2017; Lundh et al. 2017; Shekelle 2018).

Even though guidelines should be based on systematic reviews, they are far from being ‘objective’, since making guideline recommendations always involves judgement, for example, regarding the strengths and limitations of the evidence or the balance of benefits and harms (Arbeitsgemeinschaft der Wissenschaftlichen Medizinischen Fachgesellschaften AWMF—Ständige Kommission Leitlinien 2012). The review of Perkin et al. shows for example that different organisations interpret the evidence differently and come to diverging recommendation statements (Perkin et al. 2020). Moreover, CPGs and FBDGs come from different political, social and economic fields, their developers have variable, but distinctive professional backgrounds, are exposed to different professional cultures, act under different economic premises and regulations. It therefore seems reasonable to expect differences in the methodological approaches to develop such guidelines and consequently also in the quality of guidelines.

Diverging recommendations and inconsistency across guidelines might decrease the confidence of health professionals as well as of patients in guidelines, in general, if the reasoning leading to a recommendation statement is not transparent and no information regarding the developmental process is provided (Institute of Medicine US Committee on Standards for Developing Trustworthy Clinical Practice Guidelines et al. 2011). However, it has not been investigated systematically whether CPGs and FBDGs on ECAP comply with methodological standards in guideline development, and if not, whether the content of the recommendations is affected by different methodologies.

The objective of this study was to systematically assess the methodological quality of CPGs and FBDGs on ECAP and CN and to assess recommendations on ECAP regarding their consistency across guidelines.

## 2 Methods

This systematic synthesis of guidelines is reported according to the PRISMA (preferred reporting for systematic reviews and meta‐analyses) guidelines (Page et al. 2021), when applicable, and a checklist is available on figshare (https://doi.org/10.6084/m9.figshare.22886672).

### 2.1 Search Strategy

We conducted a comprehensive search for national and international CPGs and FBDGs concerning ECAP and CN according to established recommendations for guideline retrieval (Cochrane Deutschland Stiftung, et al. 2019) between February and June 2020. If available, the following search filters were applied: only fully published guidelines, published from the year 2010 onwards, and published in English or German. More information about the included databases, websites and the search strategy is available in the published study protocol (Sieferle, Schaefer, and Bitzer 2022) and a detailed list of databases and (supra)national institutions included in the search can be found in Supporting Information S1: Tables A1 and A2.

### 2.2 Eligibility Criteria

Eligible were all guidelines on ECAP and CN that included recommendations concerning allergy prevention, with pregnant women or breastfeeding women or infants (up to 1 year, with or without increased risk for the development of allergies or asthma) as the target population. The guidelines had to have a publication date from 2010 and be valid at the time of search. Eligible were guidelines with recommendations on primary preventive interventions to decrease the onset of IgE‐mediated allergies, including atopic eczema, food allergies or asthma. We considered only CPGs and FBDGs, on a national or international level, and in English or German.

Two study group members screened the retrieved records independently for their relevance according to the eligibility criteria and resolved any disagreements by discussion with the study group until consensus was reached.

### 2.3 Data Extraction

The first author extracted basic data, which was cross‐checked by another study group member. This included, among others: guideline title, first author, year of publication, country/scope, topic of the guideline, leading scientific societies, composition of guideline panel and document type (CPG, FBDG). Extracted data was entered into a relational database.

### 2.4 Quality Appraisal of Guidelines

Two reviewers determined the quality of the included guidelines using the Appraisal of Guidelines for Research and Evaluation II (AGREE II) instrument (AGREE Next Steps Consortium 2017; Brouwers et al. 2010). Both assessors used the online training tools provided by the AGREE collaboration before conducting the assessment. Additionally, after the quality assessment of the first two guidelines, the assessors compared their results and discussed the differences which emerged in the assessment. With the support of a third researcher, the overall understandings of the items of the AGREE II instrument were discussed to reach a consensus of the AGREE items and the assessment of the first two guidelines. AGREE II evaluates guideline methodology and quality and consists of 23 items covering six domains: (1) Scope and purpose, (2) Stakeholder involvement, (3) Rigour of development, (4) Clarity of presentation, (5) Applicability and (6) Editorial independence. Additionally, two Overall Guideline Assessment items are included: (1) A score from 1 to 7, indicating the general quality of the guideline and (2) the decision, whether the assessor would recommend the guideline for use in practice. This ‘requires the user to make a judgement as to the quality of the guideline, taking into account the criteria considered in the assessment process’ (Brouwers et al. 2010, p. 13). Each item of the AGREE II instrument is scored on a 7‐point scale (1 = strongly disagree to 7 = strongly agree). We calculated a quality score for each domain, by summing up the scores of the individual items per domain and scaling the obtained score as a percentage of the maximum possible score for that domain:

$$\frac{\mathrm{Obtained}\;\mathrm{score}-\mathrm{Minimum}\;\mathrm{possible}\;\mathrm{score}}{\mathrm{Maximum}\;\mathrm{possible}\;\mathrm{score}-\mathrm{Minimum}\;\mathrm{possible}\;\mathrm{score}}.$$

A higher domain score indicates a higher quality of the guideline in this domain. The six domain scores are independent and not aggregated into a single quality score (Brouwers et al. 2010).

We resolved discrepancies between the two reviewers in the scores of the final assessment by averaging the points if the scores differed by ≤ 1 point, and by discussion until consensus was reached, if scores differed by two points or more.

### 2.5 Content Analysis of Guideline Recommendations

We conducted a document analysis of relevant recommendation statements regarding ECAP. We derived codes for topics and time of intervention inductively from emerging themes in the guidelines, codes for the direction of recommendation, strength of recommendation (SoR) and level of evidence (LoE) were developed deductively based on the GRADE approach and adapted (Schünemann et al. 2013). Gradings for SoR and LoE from guidelines that used a different scale for grading were translated into our categories of weak, moderate and strong recommendations, as well as weak, moderate and high LoE.

We grouped the topic of the interventions into five inductively derived groups: (1) nutrition interventions regarding the child, (2) nutrition interventions regarding the mother, (3) interventions regarding the environment, (4) medication and emollients and (5) other interventions. For the most frequent subgroup of recommendations, the introduction of complementary foods and common allergens, we went deeper and compared the guidelines on the level of single recommendation statements regarding the direction and strength of the recommendation and the LoE.

Extraction of data and categorising and coding of recommendations was conducted in MAXQDA.

### 2.6 Data Storing and Data Management

We built a relational database containing data from the basic extraction, quality appraisal and content analysis (MS Access). All databases and the software used are stored on secure servers of the University of Education Freiburg. All involved project members are bound by the Data Protection Act (DS‐GVO) and subjected to confidentiality during the entire project phase and beyond.

### 2.7 Data Analysis

We summarised the general characteristics of the included guidelines using descriptive statistics. Quality scores for each AGREE II domain are presented using mean and standard deviation (SD). We conducted a *t*‐test for independent samples to compare the mean domain scores for CPGs and FBDGs and a Mann–Whitney *U*‐test for the variables that were not normally distributed. Statistical analysis of the extracted data was carried out with IBM SPSS Statistics software version 28.0.1.1 (14).

## 3 Results

### 3.1 Included Guidelines

**Figure 1:**
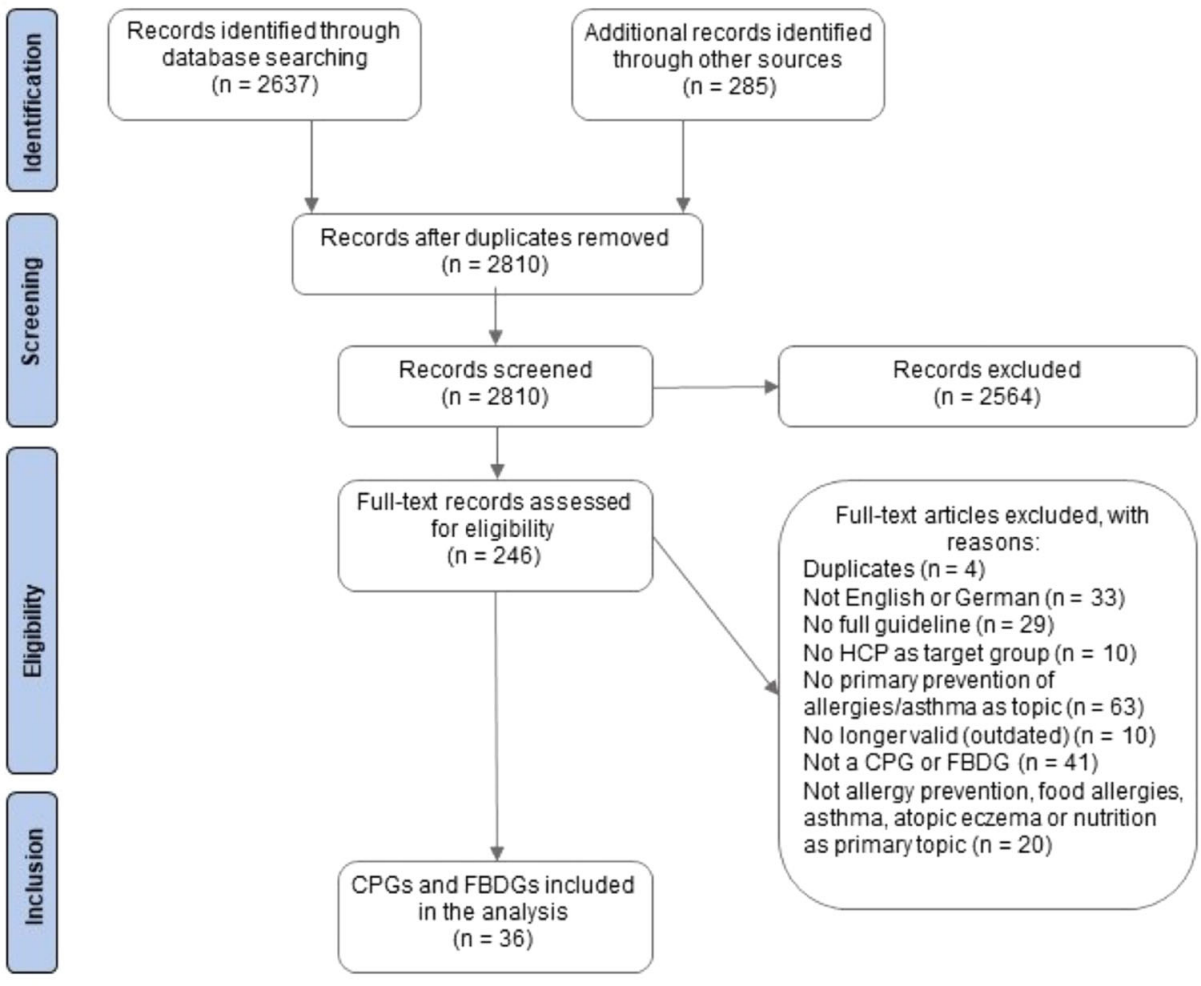
Flow‐chart of the records screened and full‐texts retrieved.

We identified a total of 2922 records and after removal of duplicates and screening of title and abstract, we excluded 2676. After full‐text screening of the remaining 246 records, 36 records were deemed eligible and included in our database (see flow‐chart in Figure 1). Of the 36 records, 23 are CPGs and 13 FBDGs. CPGs target a wider audience, with three CPGs published on international and two on European level, whereas included FBDGs are only published on national level (in *n* = 7 countries).

CPGs also cover a wider variety of topics, with nine guidelines addressing food allergies, six atopic eczema, five allergy prevention, in general, and three guidelines asthma. FBDGs are more homogeneous, with most FBDGs addressing CN (*n* = 12) and only one directly addressing food allergies (for details see Supporting Information S1: Tables A.3 and A.4).

### 3.2 Guideline Quality

**Figure 2:**
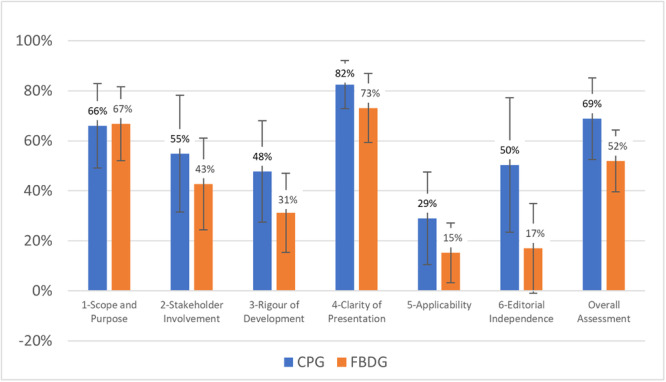
Comparison of standardised domain scores between CPGs and FBDGs (means and standard deviation).

Overall, the 36 guidelines score highest in the domains 4 ‘Clarity of Presentation’ and 1 ‘Scope and Purpose’ (79% and 66% respectively), and lowest in domains 5 ‘Applicability’ and 6 ‘Editorial Independence’ (24% and 38% respectively). The overall quality of the guidelines (AGREE Item ‘Overall Guideline Assessment—1. Rate the overall quality of this guideline’) is 63%. Comparing CPGs and FBDGs, CPGs achieve almost consistently higher scores than FBDGs. We observe the largest difference in domain 6 ‘Editorial Independence’ (Δ 33 points, 50% vs. 17%, *p* = 0.001), followed by domain 3 ‘Rigor of development’ (Δ 17 points; 48% vs. 31%, *p* = 0.016). For details see Figure 2, for results by guideline and for the differences between CPGs and FBDGs in the other domains, see Supporting Information S1: Table A.4.

### 3.3 Interventions Addressed in Guideline Recommendation Statements

The 36 guidelines contain 287 recommendations regarding ECAP. Most recommendations address nutrition interventions for the child or mother (176 rs. 49). The most frequently addressed intervention is the introduction of complementary foods and common allergens (*n* = 70), followed by breastfeeding, maternal diet during pregnancy/lactation and breast milk substitutes and formula (*n* = 37, *n* = 34 and *n* = 32, respectively). Recommendations regarding the environment included recommendations regarding mould and dampness, pet ownership, house dust mites, motor vehicle emissions, nonspecific immunomodulation, indoor air quality and exposure to tobacco smoke, whereas recommendations on medication and emollients included recommendations on antibiotics, vaccination, immunotherapy, pharmaceuticals, like paracetamol, and emollients. Other recommendations included, for example, psychosocial factors and the mode of delivery (vaginal delivery). For details, see Table 1.

**Table 1:** Interventions regarding allergy prevention in 23 CPGs and 13 FBDGs for children at average and high risk for allergies.

	Guidelines addressing the intervention		Recommendations per guideline		
	CPG	FBDG	CPG	FBDG	Recommendations per intervention
	*n*	*n*	Mean	Mean	Total
1. Nutrition interventions regarding the child	22	11	5.6	4.6	176
Introduction of complementary foods and common allergens	20	10	2.4	2.3	70^c^
Breastfeeding	17	6	1.2	2.5	37
Breast milk substitutes and formula	12	5	1.6	2.2	32
Supplementation of pre‐ and probiotics	8	2	1.8	1	17
Body weight/obesity	5		1.2		6
Vitamin D supplementation	4		1.3		5
Other supplements^a^	3		1.7		5
Child diet	4		1.3		5
2. Nutrition interventions regarding the mother	18	8	2.2	1.4	49
Maternal diet during pregnancy/lactation^b^	17	8	1.4	1.4	34
Supplementation of pre‐ and probiotics	5		1.6		8
Vitamin D supplementation	2		2		4
Other supplements^a^	3		1		3
Body weight/obesity	1		1		1
3. Environment	7	2	4.7	2.0	37
4. Medication and emollients	8	1	1.5	1.0	13
5. Other interventions	8	1	1.3	1.0	12

^a^ For Example, fish oil, n3‐fatty acids, vitamins.

^b^ Including food avoidance and specific diet.

^c^ Recommendations for children with average risk of allergies: *n* = 60, for high‐risk children: *n* = 16.

CPGs cover a wider range of ECAP interventions compared to FBDGs, and often provide more recommendations per intervention and guideline. For interventions in the child, nearly all CPGs and FBDGs address the introduction of complementary foods and common allergens, approximately half of the guidelines address breast milk substitutes and formula, whereas child body weight, Vitamin D and other supplements are covered in CPGs only. Eighteen of 23 CPGS (and 8 of 13 FBDGs) address interventions targeted at the mother, usually maternal diet during pregnancy/lactation (see Table 1). A third of the CPGs, but only two FBDGs include recommendations on interventions regarding the environment and medication (FBDGs make recommendations on pet ownership, motor‐vehicle emissions, vaccination [Koletzko et al. 2016] and exposure to tobacco smoke [Koletzko et al. 2016; Schweizerische Gesellschaft für Ernährung 2019]). The average number of recommendations per guideline on a given intervention varies between CPGs and FBDGs depending on the type of intervention. CPGs provide more recommendations on introduction of complementary foods and common allergens, maternal interventions and environmental interventions, whereas FBDGs offered more recommendations on breastfeeding and breast milk substitution (see Table 1).

### 3.4 Recommendations on the Most Commonly Addressed Intervention: Introduction of Complementary Foods and Common Allergens

Do CPGs and FBDGs agree on their recommendations on interventions covered in both types of guidelines? Of 70 single recommendations on the introduction of complementary foods and common allergens (weaning) 60 are directed at children at average risk for allergies, 16 recommendations address high‐risk children (six recommendations address both groups). We condensed the 60 recommendations on weaning for average risk children to 26 content identical recommendations (see Table 2, Guideline abbreviations are defined in Supporting Information S1: Table A.3) (see Supporting Information S1: Table A.5 for recommendations for high‐risk children, Guideline abbreviations are defined in Supporting Information S1: Table A.3).

**Table 2:** Twenty‐six content identical recommendation statements regarding the introduction of complementary foods and common allergens in CPGs and FBDGs for average risk children (direction and strength of recommendation and level of evidence).

	Recommended		Not recommended		No recommendation			
Recommendation statement	CPG (Abbreviation)	FBDG (Abbreviation)	CPG (Abbreviation)	FBDG (Abbreviation)	CPG (Abbreviation)	FBDG (Abbreviation)	SoR	LoE
1. Introducing solid foods at 4–6 months of age	EDF 2018						++	++
	Chan 2016							
	AMS 2010						+++	+++
	JSPACI 2017							
	SIPPS 2016							
	EAACI 2014						+	++
		NGIL 2016						
		THL 2019						
		ÖGKJ 2010						
		SGE 2019						
					CPS 2013			++
						AHS 2015		
2. Introducing solid foods at 6 months of age	SIGN 2011							++
3. Introducing common allergens before 4 months of age					DGAKI 2014			
4. Introducing common allergens at 4–6 months of age	Chan 2016							
	SIPPS 2016							
	NIAID 2010						+	+
		Perez‐Escamilla 2017						
5. Introducing common allergens at 6 months of age		HC 2015						
		HC 2014						
6. Early introduction of common allergens					BSACI 2010			
					SIGN 2019			
7. Introducing fish at 4–6 months of age	DGAKI 2014						++	++
		ÖGKJ 2010						++
8. Introducing fish at 6–9 months of age	Recto 2017						+	++
9. Introducing egg at 4–6 months of age	Recto 2017						+	+
		ÖGKJ 2010						++
10. Introducing wheat at 4–6 months of age	Recto 2017						+	++
		ÖGKJ 2010						
11. Introducing peanut when age and appropriate	NIAID 2017						+	+
12. Introducing peanut at 6 months of age		NHMRC 2013						
13. Introducing peanut at 4–11 months of age	Recto 2017						+++	+++
14. Early introduction of peanut					AAP 2019			
15. Delayed introduction of peanut					BSACI 2017			
16. Delayed introduction of solids beyond 4 months of age			DGAKI 2014				+++	++
17. Delayed introduction of solids			NIAID 2010				+	
beyond 4–6 months of age			JSPACI 2017					
			AMS 2010				+++	++
18. Delayed introduction of solids, including common allergens, beyond 6 months of age			CPS 2013					++
			Recto 2017				+++	++
						NHMRC 2012		
						NHMRC 2013		
19. Delayed introduction of common allergens beyond 4–6 months of age			JSPACI 2017					
			EAACI 2014				+	++
				HC 2015				
				ÖGKJ 2010				++
					AAP 2019			
20. Avoidance of common allergens			AAAAI 2014				+	++
during weaning			SIGN 2019				+++	+++
			DGAKI 2014				++	
				ÖGKJ 2010			++	
				SGE 2019				++
					CDA 2019			
						NHMRC 2012		
21. Avoidance of acidic foods				Perez‐Escamilla 2017				
22. Introduce a diversity of foods during weaning	EDF 2018						+	+++
23. Introduce peanut cautiously at home	BSACI 2017							
24. Introduce other solids before peanut, to ensure developmental readiness	NIAID 2017							
25. For introduction of solids, take developmental readiness, nutritional needs, and so on into consideration	Chan 2016							
26. Feed newly introduced solids regularly, to maintain tolerance	CPS 2013							++

*Note:* + weak recommendation/low level of evidence; ++ moderate recommendation/moderate level of evidence; +++ strong recommendation/high level of evidence. Abbreviations of guidelines are explained in Supporting Information S1: Table A.3.

Abbreviations: LoE, level of evidence; SoR, strength or recommendation.

Information on the LoE is available in 25 of 60 recommendations (42%), 20 are accompanied by a grading of the SoR (33%) and 17 of 60 recommendations (28%) are presented with LoE and SoR.

Most guidelines agree on the introduction of complementary foods: they recommend introducing solids between 4 and 6 months of age, and/or advice against delaying the introduction beyond that age. Two guidelines, published in 2015 and 2013 (Alberta Health Services 2015, p. 4.1.4; Chan et al. 2013, p. 547), make no recommendation on the appropriate timing yet, because ‘inducing tolerance by introducing solid foods at 4–6 months of age is currently under investigation and cannot be recommended at this time’, and one guideline, published 2011, recommends introducing solids only at 6 months of age ‘Current UK guidelines based on recommendations from the WHO recommend that weaning should start at 6 months’ (Scottish Intercollegiate Guidelines Network [SIGN] 2011, p. 19).

There is slightly less agreement regarding the introduction of common allergens in general: among the 20 recommendations, some focus on specific timepoints of introduction, some advise for early or against a delayed introduction. Guidelines vary in recommending the introduction between 4 and 6 months of age (Chan et al. 2016; Di Mauro et al. 2016; NIAID‐Sponsored Expert Panel 2010; Pérez‐Escamilla, Segura‐Pérez, and Lott 2017), after 6 months (Health Canada et al. 2014, 2015), and two guidelines refrain explicitly from a recommendation but do not provide formal assessment of LoE or SoR (Clark et al. 2010; Scottish Intercollegiate Guidelines Network [SIGN] and British Thoracic Society 2019). The few recommendations that address the introduction of single allergens, for example, peanut, egg, wheat and fish, do have nuanced differences.

By visual inspection of the table, we could not find a convincing association between the content of the recommendations and the use of SoR or LoE ratings.

The much shorter table on recommendations directed at high‐risk children depicts a similar picture: more or less nuanced discrepancies between CPGs and FBDGs and no apparent association between SoR or LoE ratings and the content of the recommendation (see Supporting Information S1: Table A.5).

## 4 Discussion

The aim of this study is to systematically assess the methodological quality of CPGs and FBDGs on ECAP and CN and to assess the consistency of recommendations on ECAP across guidelines. Our expectation is to obtain a comprehensive understanding of the methodological rigour employed in the guideline development, to identify their strengths and weaknesses and to explore the content of their recommendations.

Our findings indicate that the quality of CPGs and FBDGs on ECAP and CN differs significantly, with especially low scores in AGREE‐domains 3 ‘Rigour of Development’, 5 ‘Applicability’ and 6 ‘Editorial Independence’. These domains focus on the methodological quality of the guideline development, including evidence search and synthesis, COI management and guideline implementation. Other studies have shown similar deficits in the methodological quality of guideline development (Bhatt et al. 2018; Chiappini et al. 2017), but not in direct comparison of guidelines from two different subject fields.

CPGs generally score higher than FBDGs, possibly because methodological standards for guideline development differ for CPGs and FBDGs and are not as widely used in FBDGs yet (EFSA Panel on Dietetic Products and Nutrition and Allergies [NDA] 2010). Differences are especially prevalent in domains 3 and 6 and could be seen as an urgent need to implement higher methodological standards in the development of FBDGs. Even though FBDGs should be based on nutrient requirements, current dietary practices and public health problems, current scientific evidence should also factor into the formulating of recommendations (Bechthold et al. 2018; World Health Organisation 1998). The low score in domain 3 (Rigour of Development) indicates that FBDGs need improvement when it comes to search and selection of evidence and the formulating of recommendations, even when acknowledging that the methods used to develop and assess CPGs are not always suitable for FBDGs, and the questions FBDGs want to answer (Bero, Norris, and Lawrence 2019). The low score in domain 6 (Editorial Independence) is concerning, as COI can emerge from industry sponsorship of the guideline or from industry ties of guideline authors. Studies have shown this risk for example concerning the marketing of commercial milk formula (Rollins et al. 2023; Pérez‐Escamilla et al. 2023). Low transparency on the formulating of recommendations, the funding of the guideline development and potential COI can lead to reduced trust in the guideline.

In our sample, the guidelines with high methodological quality according to AGREE II were developed by organisations such as the AWMF, SIGN, GINA, AAD, NIAID and EAACI (Schäfer et al. 2014; Scottish Intercollegiate Guidelines Network [SIGN] and British Thoracic Society 2019; Global Initiative for Asthma 2020; Scottish Intercollegiate Guidelines Network [SIGN] 2011; Eichenfield et al. 2013; NIAID‐Sponsored Expert et al. 2010; Muraro et al. 2014). These medical organisations are often very active in the scientific development and testing of methods to produce high‐quality guidelines, have published detailed guidance on guideline development or use the tools GRADE (Schünemann et al. 2013) and AGREE II (AGREE Next Steps Consortium 2017), therefore ensuring a higher methodological standard.

Although the methodological quality varies considerably across the guidelines, we were surprised to find that the recommendation statements exhibit only minor, in some cases rather nuanced discrepancies. Most guidelines recommend introducing complementary foods, including common allergens, between 4 and 6 months of age, and advise against delaying the introduction of any food items. However, a few guidelines make other recommendations, for example, the SIGN guideline on the management of atopic eczema from 2011, which indirectly recommends the introduction of complementary foods at 6 months (Scottish Intercollegiate Guidelines Network [SIGN] 2011). The observed minor differences in recommendations may partly be explained by the year of publication and the evidence used and availability at that time.

Only a quarter of the recommendation statements were given a formal grading of the SoR and LoE, usually attributed to higher methodological rigour and quality. However, this turned out not to be relevant when it came to the question ‘What to recommend?’, since we saw no convincing associations between those gradings and the content of the recommendations.

How did FBDGs reach recommendations quite similar to CPGs despite their significantly lower methodological quality? Is the higher methodological standard even necessary to provide clear recommendations on ECAP? We caution against such a conclusion for several reasons: (1) It might be the case in ECAP that the paradigm shift from avoidance of allergens to early and sustained exposure has gained widespread acceptance among clinicians and nutritionists. Then, providing guideline recommendations on ECAP would simply reflect common knowledge, without the need to prove the evidence again (like buckling up in cars). But how would we (or a guideline panel) assess the level of ‘unity’, ‘common knowledge’ or ‘controversy’ to decide for or against a more elaborate methodological process? (2) Our in‐depth assessment of the recommendation statements focused solely on the topic of introduction of complementary foods and common allergens. For other ECAP interventions, such as formula and breast milk substitutes or pre‐ and probiotic supplementation, the situation could be much different. Lower methodological quality, especially lack of information on COI management, as reflected in AGREE‐domain 6, may play a substantial role here (World Health Organization 2018; Rollins et al. 2023). (3) Our study indicates that it takes a lot of resources, efforts and manpower to check if guidelines developed with lower quality recommend roughly the same as higher quality guidelines. So even if lower quality guideline recommendations do not differ substantially from high‐quality guidelines, low methodological quality and little transparency can lead to distrust in the recommendations and hinder the application of the guideline in clinical/extraclinical practice (Institute of Medicine US Committee on Standards for Developing Trustworthy Clinical Practice Guidelines et al. 2011). Studies have shown low rates of guideline implementation and low levels of adherence to the recommendations, for example, peanut allergy, leading to suboptimal healthcare provision (Gupta et al. 2020; Hoffman et al. 2017). Improvements in guideline methodology could increase trust in the guideline as well as dissemination of the guideline, improve implementation and adherence to the recommendations, and consequently reduce the incidence of allergic diseases in infants.

We would like to point at another issue: AGREE II does assess the rigour of development, which includes the search for evidence and formulating of the recommendations, but does not take the evidence base of the recommendations in detail into account. A close look on the type of sources used to justify the recommendation statements on ECAP would be interesting and worthwhile. To explore if guidelines with varying methodological quality and management of COI use similar evidence (i.e., systematic reviews, high‐quality primary studies) would be a logical next step.

## 5 Conclusions

In conclusion, this investigation highlights deficits in guideline development, especially in FBDGs. Although the recommendations concerning the introduction of complementary foods in our sample show low variability, since childhood allergies are an important public health concern, and effective allergy prevention in early childhood is available, feasible but not fully implemented on population level, a higher methodological quality of guidelines concerning ECAP should be promoted to increase confidence in the recommendations.

## Author Contributions

**Eva M. Bitzer:** conceptualisation, funding acquisition, methodology, project administration, supervision, writing–review and editing. **Katharina Sieferle:** conceptualisation, data curation, formal analysis, methodology, visualisation, writing–original draft, writing–review and editing.

## Conflicts of Interest

The authors declare no conflicts of interest.

## Supporting information

Supporting information.

## Data Availability

The data that support the findings of this study are available on request from the corresponding author.

## References

[mcn13779-bib-0001] AGREE Next Steps Consortium . 2017. *The AGREE II Instrument (Electronic Version)*. http://www.agreetrust.org.

[mcn13779-bib-0002] Alberta Health Services . 2015. *Nutrition Guideline Healthy Infants and Young Children Introduction of Complementary Foods*. https://www.albertahealthservices.ca//nutrition/Page8567.aspx.

[mcn13779-bib-0003] Arbeitsgemeinschaft der Wissenschaftlichen Medizinischen Fachgesellschaften (AWMF)—Ständige Kommission Leitlinien . 2012. *AWMF‐Regelwerk “Leitlinien”. 1. Auflage 2012*. http://www.awmf.org/leitlinien/awmf-regelwerk.html.

[mcn13779-bib-0004] Bantz, S. K. , Z. Zhu , and T. Zheng . 2014. “The Atopic March: Progression From Atopic Dermatitis to Allergic Rhinitis and Asthma.” Journal of Clinical & Cellular Immunology 5, no. 2: 202. 10.4172/2155-9899.1000202. 25419479 PMC4240310

[mcn13779-bib-0005] Bechthold, A. , H. Boeing , I. Tetens , L. Schwingshackl , and U. Nöthlings . 2018. “Perspective: Food‐Based Dietary Guidelines in Europe—Scientific Concepts, Current Status, and Perspectives.” Advances in Nutrition 9, no. 5: 544–560. 10.1093/advances/nmy033. 30107475 PMC6140433

[mcn13779-bib-0006] Bero, L. A. , S. L. Norris , and M. A. Lawrence . 2019. “Making Nutrition Guidelines Fit for Purpose.” BMJ 365: l1579. 10.1136/bmj.l1579. 30992263

[mcn13779-bib-0007] Bhatt, M. , A. Nahari , P. W. Wang , et al. 2018. “The Quality of Clinical Practice Guidelines for Management of Pediatric Type 2 Diabetes Mellitus: A Systematic Review Using the Agree II Instrument.” Systematic Reviews 7, no. 1: 193. 10.1186/s13643-018-0843-1. 30442196 PMC6238336

[mcn13779-bib-0008] Bindslev, J. B. B. , J. Schroll , P. C. Gøtzsche , and A. Lundh . 2013. “Underreporting of Conflicts of Interest in Clinical Practice Guidelines. Cross Sectional Study.” BMC Medical Ethics 14: 19. 10.1186/1472-6939-14-19. 23642105 PMC3651727

[mcn13779-bib-0010] Brouwers, M. C. , M. E. Kho , G. P. Browman , et al. 2010. “AGREE II: Advancing Guideline Development, Reporting and Evaluation in Healthcare.” Canadian Medical Association Journal 182: E839–E842. 10.1503/cmaj.090449. 20603348 PMC3001530

[mcn13779-bib-0011] Chan, A. W. M. , J. K. C. Chan , A. Y. C. Tam , T. F. Leung , and T. H. Lee . 2016. “Guidelines for Allergy Prevention in Hong Kong.” Hong Kong Medical Journal = Xianggang yi xue za zhi 22, no. 3: 279–285. 10.12809/hkmj154763. 27305695

[mcn13779-bib-0012] Chan, E. S. , and C. Cummings , Canadian Paediatric Society, Community Paediatrics Committee, and Allergy Section . 2013. “Dietary Exposures and Allergy Prevention in High‐Risk Infants. A Joint Statement With the Canadian Society of Allergy and Clinical Immunology.” Paediatrics & Child Health 18 10: 545–554. 10.1093/pch/18.10.545. 24497783 PMC3907352

[mcn13779-bib-0013] Chiappini, E. , B. Bortone , L. Galli , and M. Martino . 2017. “Guidelines for the Symptomatic Management of Fever in Children: Systematic Review of the Literature and Quality Appraisal With AGREE II.” BMJ Open 7, no. 7: e015404. 10.1136/bmjopen-2016-015404. PMC5642818 28760789

[mcn13779-bib-0014] Clark, A. T. , I. Skypala , S. C. Leech , et al. 2010. “British Society for Allergy and Clinical Immunology Guidelines for the Management of Egg Allergy.” Clinical & Experimental Allergy 40, no. 8: 1116–1129. 10.1111/j.1365-2222.2010.03557.x. 20649608

[mcn13779-bib-0009] Cochrane Deutschland Stiftung, Institut für Evidenz in der Medizin, Institut für Medizinische Biometrie und Statistik, Freiburg, Arbeitsgemeinschaft der Wissenschaftlichen Medizinischen Fachgesellschaften ‐ Institut für Medizinisches Wissensmanagement, Ärztliches Zentrum für Qualität in der Medizin . 2019. “Manual: Systematische Recherche für Evidenzsynthesen und Leitlinien.” Version 2.1. Auflage (01.04.2019). Verfügbar: Cochrane Deutschland: https://www.cochrane.de/de/literaturrecherche; AWMF: https://www.awmf.org/leitlinien/awmf-regelwerk/ll‐entwicklung.html; ÄZQ: https://www.aezq.de/aezq/publikationen/azq‐partner#literaturrecherche. https://freidok.uni‐freiburg.de/data/149324.

[mcn13779-bib-0015] Dierick, B. , T. van der Molen , B. Flokstra‐de Blok , et al. 2020. “Burden and Socioeconomics of Asthma, Allergic Rhinitis, Atopic Dermatitis and Food Allergy.” Expert Review of Pharmacoeconomics & Outcomes Research 20, no. 5: 437–453. 10.1080/14737167.2020.1819793. 32902346

[mcn13779-bib-0016] Di Mauro, G. , R. Bernardini , S. Barberi , et al. 2016. “Prevention of Food and Airway Allergy: Consensus of the Italian Society of Preventive and Social Paediatrics, the Italian Society of Paediatric Allergy and Immunology, and Italian Society of Pediatrics.” World Allergy Organization Journal 9: 28. 10.1186/s40413-016-0111-6. 27583103 PMC4989298

[mcn13779-bib-0017] Eccles, M. P. , J. M. Grimshaw , P. Shekelle , H. J. Schünemann , and S. Woolf . 2012. “Developing Clinical Practice Guidelines: Target Audiences, Identifying Topics for Guidelines, Guideline Group Composition and Functioning and Conflicts of Interest.” Implementation Science 7, no. 1: 60. 10.1186/1748-5908-7-60. 22762776 PMC3523009

[mcn13779-bib-0018] EFSA Panel on Dietetic Products and Nutrition and Allergies (NDA) . 2010. “Scientific Opinion on Establishing Food‐Based Dietary Guidelines.” EFS2 8, no. 3: 42. 10.2903/j.efsa.2010.1460.

[mcn13779-bib-0019] Eichenfield, L. F. , W. L. Tom , S. L. Chamlin , et al. 2013. “Guidelines of Care for the Management of Atopic Dermatitis: Section 1. Diagnosis and Assessment of Atopic Dermatitis.” Journal of the American Academy of Dermatology 70, no. 2: 338–351. 10.1016/j.jaad.2013.10.010. 24290431 PMC4410183

[mcn13779-bib-0020] Global Initiative for Asthma . 2020. *Global Strategy for Asthma Management and Prevention*. www.ginasthma.org.

[mcn13779-bib-0021] Gupta, R. S. , L. A. Bilaver , J. L. Johnson , et al. 2020. “Assessment of Pediatrician Awareness and Implementation of the Addendum Guidelines for the Prevention of Peanut Allergy in the United States.” JAMA Network Open 3, no. 7: e2010511. 10.1001/jamanetworkopen.2020.10511. 32667655 PMC7364336

[mcn13779-bib-0022] Hansen, C. , A. Lundh , K. Rasmussen , P. C. Gøtzsche , and A. Hróbjartsson . 2017. “Financial Conflicts of Interest and Outcomes and Quality of Systematic Reviews.” Cochrane Database of Systematic Reviews 365, no. 9465: 1159. 10.1002/14651858.MR000047. PMC7040976 31425611

[mcn13779-bib-0023] Health Canada, Canadian Paediatric Society, Dietitians of Canada, and Breastfeeding Committee for Canada . 2014. *Nutrition for Healthy Term Infants: Recommendations From 6 to 24 Months*. https://www.canada.ca/en/health-canada/services/canada-food-guide/resources/infant-feeding/nutrition-healthy-term-infants-recommendations-birth-six-months/6-24-months.html. 10.3148/73.4.2012.204 23217450

[mcn13779-bib-0024] Health Canada, Canadian Paediatric Society, Dietitians of Canada, and Breastfeeding Committee for Canada . 2015. *Nutrition for Healthy Term Infants: Recommendations From Birth to Six Months*. https://www.canada.ca/en/health-canada/services/canada-food-guide/resources/infant-feeding/nutrition-healthy-term-infants-recommendations-birth-six-months.html#a4. 10.3148/73.4.2012.204 23217450

[mcn13779-bib-0025] Hoffman, B. , L. Moreno , L. Gerber , D. D'Angelo , and E. Abramson . 2017. “OR075 What Pediatricians Are Advising on Infant Peanut Introduction.” Annals of Allergy, Asthma & Immunology 119, no. 5: S10. 10.1016/j.anai.2017.08.057.

[mcn13779-bib-0026] Institute of Medicine (US) Committee on Standards for Developing Trustworthy Clinical Practice Guidelines , R. Graham , M. Mancher , D. Miller Wolman , S. Greenfield , and E. Steinberg . 2011. Clinical Practice Guidelines We Can Trust. Washington, DC: National Academies Press. https://www.ncbi.nlm.nih.gov/books/NBK209539/. 24983061

[mcn13779-bib-0027] Koletzko, B. , C. P. Bauer , M. Cierpka , et al. 2016. “Ernährung und Bewegung von Säuglingen und stillenden Frauen. Aktualisierte Handlungsempfehlungen von ‘Gesund ins Leben—Netzwerk Junge Familie’, eine Initiative von IN FORM.” Monatsschrift Kinderheilkunde 164, no. S5: 433–457. https://www.gesund-ins-leben.de/.

[mcn13779-bib-0028] Lundh, A. , J. Lexchin , B. Mintzes , J. B. Schroll , and L. Bero . 2017. “Industry Sponsorship and Research Outcome.” Cochrane Database of Systematic Reviews 2: 000033. 10.1002/14651858.MR000033.pub3. PMC8132492 28207928

[mcn13779-bib-0029] Montagnese, C. , L. Santarpia , M. Buonifacio , et al. 2015. “European Food‐Based Dietary Guidelines. A Comparison and Update.” Nutrition 31, no. 7–8: 908–915. 10.1016/j.nut.2015.01.002. 26015390

[mcn13779-bib-0030] Muraro, A. , S. Halken , S. H. Arshad , et al. 2014. “EAACI Food Allergy and Anaphylaxis Guidelines. Primary Prevention of Food Allergy.” Allergy 69, no. 5: 590–601. 10.1111/all.12398. 24697491

[mcn13779-bib-0031] NIAID‐Sponsored Expert Panel . 2010. “Guidelines for the Diagnosis and Management of Food Allergy in the United States: Report of the NIAID‐Sponsored Expert Panel.” Journal of Allergy and Clinical Immunology 126, no. 6 Suppl: 1–58. 10.1016/j.jaci.2010.10.007. PMC4241964 21134576

[mcn13779-bib-0032] Page, M. J. , J. E. McKenzie , P. M. Bossuyt , et al. 2021. “The PRISMA 2020 Statement: An Updated Guideline for Reporting Systematic Reviews.” PLoS Medicine 18, no. 3: e1003583. 10.1371/journal.pmed.1003583. 33780438 PMC8007028

[mcn13779-bib-0033] Pawankar, R. 2014. “Allergic Diseases and Asthma. A Global Public Health Concern and a Call to Action.” World Allergy Organization Journal 7, no. 1: 12. 10.1186/1939-4551-7-12. 24940476 PMC4045871

[mcn13779-bib-0034] Pawankar, R. , G. W. Canonica , T. Holgate Stephen , R. F. Lockey , and M. S. H. Blaiss. 2013. *The WAO White Book on Allergy (Update 2013)*. Milwaukee, WI, USA: World Allergy Organization.

[mcn13779-bib-0035] Pérez‐Escamilla, R. , S. Segura‐Pérez , and M. Lott . 2017. “Feeding Guidelines for Infants and Young Toddlers: A Responsive Parenting Approach.” Nutrition Today 52, no. 5: 223–231. 10.1097/NT.0000000000000234.

[mcn13779-bib-0036] Pérez‐Escamilla, R. , C. Tomori , S. Hernández‐Cordero , et al. 2023. “Breastfeeding: Crucially Important, but Increasingly Challenged in a Market‐Driven World.” Lancet 401, no. 10375: 472–485. 10.1016/S0140-6736(22)01932-8. 36764313

[mcn13779-bib-0037] Perkin, M. R. , A. Togias , J. Koplin , and S. Sicherer . 2020. “Food Allergy Prevention: More Than Peanut.” Journal of Allergy and Clinical Immunology 8, no. 1: 1–13. 10.1016/j.jaip.2019.11.002. 31950900

[mcn13779-bib-0038] Qaseem, A. 2012. “Guidelines International Network. Toward International Standards for Clinical Practice Guidelines.” Annals of Internal Medicine 156, no. 7: 525. 10.7326/0003-4819-156-7-201204030-00009. 22473437

[mcn13779-bib-0039] Rollins, N. , E. Piwoz , P. Baker , et al. 2023. “Marketing of Commercial Milk Formula: A System to Capture Parents, Communities, Science, and Policy.” Lancet 401, no. 10375: 486–502. 10.1016/S0140-6736(22)01931-6. 36764314

[mcn13779-bib-0040] Schäfer, T. , C.‐P. Bauer , K. Beyer , et al. 2014. *S3‐Leitlinie Allergieprävention Update 2014*. Leitlinie der Deutschen Gesellschaft für Allergologie und klinische Immunologie (DGAKI) und der Deutschen Gesellschaft für Kinder‐ und Jugendmedizin (DGKJ). https://www.awmf.org.

[mcn13779-bib-0041] Schünemann, H. , J. Brożek , G. Guyatt , and A. Oxman . 2013. *GRADE Handbook: Introduction to GRADE Handbook for Grading the Quality of Evidence and the Strength of Recommendations Using the GRADE Approach*. Hg. v. GRADE Working Group. https://gdt.gradepro.org/app/handbook/handbook.html.

[mcn13779-bib-0042] Schweizerische Gesellschaft für Ernährung . 2019. *Ernährung des Säuglings im ersten Lebensjahr*. http://www.sge-ssn.ch/media/Merkblatt_Ernaehrung_des_Saeuglings_im_ersten_Lebensjahr-2019.pdf.

[mcn13779-bib-0043] Scottish Intercollegiate Guidelines Network (SIGN) . 2011. *Management of Atopic Eczema in Primary Care. A National Clinical Guideline (SIGN Guideline No 125)*. Edinburgh. http://www.sign.ac.uk.

[mcn13779-bib-0044] Scottish Intercollegiate Guidelines Network (SIGN) and British Thoracic Society . 2019. *British Guideline on the Management of Asthma. A National Clinical Guideline (SIGN Guideline No 158)*. Edinburgh. http://www.sign.ac.uk.

[mcn13779-bib-0045] Shekelle, P. G. 2018. “Clinical Practice Guidelines. What's Next?” Journal of the American Medical Association 320, no. 8: 757. 10.1001/jama.2018.9660. 30098168

[mcn13779-bib-0046] Sieferle, K. , C. Schaefer , and E. M. Bitzer . 2022. “Management of Evidence and Conflict of Interest in Guidelines on Early Childhood Allergy Prevention and Child Nutrition: Study Protocol of a Systematic Synthesis of Guidelines and Explorative Network Analysis.” F1000Research 11: 1290. 10.12688/f1000research.123571.2. 38239264 PMC10794862

[mcn13779-bib-0047] World Health Organization . 1998. *Preparation and Use of Food‐Based Dietary Guidelines*. Report of a Joint FAO/WHO Consultation. Geneva: WHO (WHO Technical Report Series, 880). 9795598

[mcn13779-bib-0048] World Health Organization . 2018. *Marketing of Breast‐Milk Substitutes: National Implementation of the International Code*. Geneva: World Health Organization.

