# Supplementary material for: Methodological Quality and Content of Guidelines on Early Childhood Allergy Prevention: A Systematic Assessment and Content Analysis
Source: Matern Child Nutr. 2024 Dec 13;21(2):e13779. doi: 10.1111/mcn.13779 (PMC11956064; doi:10.1111/mcn.13779)
Supplement: Supplementary file 1 — Supporting information. [file MCN-21-e13779-s001.docx]

# APPENDIX A

Table A. 1 Food and Agriculture Organization of the United Nations (FAO) directory for institutions reporting on FBDGs

| Europe | Institutional contacts |
| --- | --- |
| Albania | [Ministry of Health](http://www.shendetesia.gov.al/) |
| Austria | [Ministry of Health](http://www.bmg.gv.at/) |
| Belgium | [Federal Public Service Health, Food Chain Safety and](http://www.health.belgium.be/eportal/index.htm) [Environment](http://www.health.belgium.be/eportal/index.htm) |
| Bosnia and Herzegovina | [Institute of Public Health of Federation of Bosnia and](http://www.zzjzfbih.ba/)  [Herzegovina](http://www.zzjzfbih.ba/) |
| Bulgaria | [Ministry of Health](http://www.mh.government.bg/bg/) |
| Croatia | [Ministry of Health](http://www.zdravlje.hr/) |
| Cyprus | [Ministry of Health](http://www.moh.gov.cy/moh/moh.nsf/index_gr/index_gr?OpenDocument) |
| Denmark | [Ministry of Food, Agriculture and Fisheries](http://www.altomkost.dk/Services/Kontakt/forside.htm) |
| Estonia | [National Institute for Health Development](http://www.toitumine.ee/trukised/?type=13997) |
| Finland | [National Nutrition Council](http://www.ravitsemusneuvottelukunta.fi/portal/en/nutrition%2Brecommendations/) |
| France | [Ministry of Health](http://social-sante.gouv.fr/) |
| Georgia | [National Centre for Disease Control and Public Health](http://www.ncdc.ge/?lang=eng) |
| Germany | [German Nutrition Society](http://www.dge.de/modules.php?name=Content&pa=showpage&pid=12) |
| Greece | [National and Kapodistrian University of Athens, School of](http://www.nut.uoa.gr./contactENG.html) [Medicine - WHO Collaborating Center for Food and Nutrition](http://www.nut.uoa.gr./contactENG.html)  [Policies](http://www.nut.uoa.gr./contactENG.html) |
| Hungary | [National Institute for Food and Nutrition Science](http://www.oeti.hu/index.php?m1id=20&m2id=207) |
| Iceland | [The Directorate of Health](http://www.landlaeknir.is/english/) |
| Ireland | [Department of Health](https://www.healthpromotion.ie/) |
| Israel | [Ministry of Health](http://www.health.gov.il/Subjects/FoodAndNutrition/Nutrition/Adequate_nutrition/Pages/default.aspx) |
| Italy | [Research Centre on Food and Nutrition](http://sito.entecra.it/portale/index2.php?lingua=IT&access_flag=0) |
| Latvia | [Ministry of Health](http://www.vm.gov.lv/) |
| Malta | [The Health Promotion and Disease Prevention Directorate,](https://ehealth.gov.mt/HealthPortal/default.aspx) [Parliamentary Secretariat for Health](https://ehealth.gov.mt/HealthPortal/default.aspx) |
| Netherlands | [Netherlands Nutrition Centre](http://www.voedingscentrum.nl/) |
| Norway | [Directorate of Health](http://www.helsedirektoratet.no/Sider/default.aspx) |
| Poland | [National Food and Nutrition Institute](http://www.izz.waw.pl/en/) |
| Portugal | [Faculty of Food Sciences and Nutrition, Porto University](http://www.fcna.up.pt/) |
| Romania | [National Food and Nutrition Committee, Ministry of Health](http://www.ms.gov.ro/?pag=1) |
| Slovenia | [National Institute of Public Health](http://www.nijz.si/) |
| Spain | [Spanish Agency For Consumer Affairs, Food Safety and Nutrition](http://aesan.msssi.gob.es/en/AESAN/web/sobre_aesan/sobre_aecosan.shtml) |
| Switzerland | [Federal Food Safety and Veterinary Office](http://www.blv.admin.ch/index.html?lang=en) [Swiss Society for Nutrition](http://www.sge-ssn.ch/de/ich-und-du/essen-und-trinken/ausgewogen/lebensmittelpyramide/) |
| The former Yugoslav Republic of Macedonia | [Institute of Public Health](http://www.iph.mk/) |
| Turkey | [Ministry of Health](http://www.saglik.gov.tr/EN/ana-sayfa/2-0/20141211.html) |
| United Kingdom | [National Health Service](http://www.nhs.uk/Livewell/Goodfood/Pages/eatwell-plate.aspx) |
| Others | |
| Canada | [Health Canada](http://www.hc-sc.gc.ca/index-eng.php) |
| United States | [United States Department of Agriculture](http://www.usda.gov/wps/portal/usda/usdahome) |
| Chile | [Institute of Nutrition and Food Technology (INTA), the University](http://www.inta.cl/) [of Chile](http://www.inta.cl/) |
| Mexico | [National Institute of Public Health](http://www.insp.mx/) |
| Australia | [National Health and Medical Research Council](http://www.nhmrc.gov.au/your-health/nutrition) |

**Source:** http://www.fao.org/nutrition/education/food-based-dietary-guidelines/en/ [08.11.18]

Table A. 2 List of relevant databases, institutions and professional associations reporting on CPGs

| Relevant databases | (international level) |
| --- | --- |
| G-I-N | Guidelines International Network’s database |
| Guideline Central |  |
| NGC | US National Guideline Clearinghouse (until 09/18) |
| Relevant institutions | and expert associations (supranational level, selected countries) |
| WHO | World Health Organization |
| Europe |  |
| EAACI | European Academy of Allergy and Clinical Immunology |
| ESPEN | European Society for Clinical Nutrition and Metabolism |
| ESPGHAN | European Society for Pediatric Gastroenterology Hepatology and Nutrition |
| NICE | National Institute for Health and Care Excellence |
| The Americas |  |
| CDC | Centers for Disease Control and Prevention |
| ODPHP | Office of Disease Prevention and Health Promotion |
| USDA | United States Department of Agriculture. Center for Nutrition Policy and Promotion |
| ASPEN | American Society for Parenteral and Enteral Nutrition |
| AAAAI | The American Academy of Allergy, Asthma & immunology |
| AHS | Alberta Health Services |
| CSACI | Canadian Society of Allergy and Clinical Immunology |
| Australia |  |
| NWS | Nutrition Society of Australia |
| Relevant institutions | and expert association (national level) |
| ABAP | German Action Alliance for Allergy Prevention (Aktionsbündnis Allergieprävention) |
| AeDA | German Medical Association of Allergologists (Ärzteverband Deutscher Allergologen) |
| ADP | German Working Group on Dermatological Prevention (Arbeitsgemeinschaft Dermatologische Prävention) |
| AK-DIDA | German Task force on Dietetics in Allergology |
| AWMF | Arbeitsgemeinschaft der Wissenschaftlichen Medizinischen Fachgesellschaften |
| BVDD | Professional Association of German Dermatologists (Berufsverband der Deutschen Dermatologen) |
| BVHNO | German Professional Association of ENT Physicians (Berufsverband der HNO- Ärzte) |
| BVKJ | German Professional Association of Pediatricians (Berufsverband der Kinder- und Jugendärzte) |
| DAAB | German Allergy and Asthma Association (Deutscher Allergie- und Asthmabund) |
| DDG | German Dermatological Society (Deutsche Dermatologische Gesellschaft) |
| DGE | German Nutrition Society (Deutsche Gesellschaft für Ernährung) |
| DGEM | German Society for Nutritional Medicine (Deutsche Gesellschaft für Ernährungsmedizin) |
| DGHNOKHC | German Society for Oto-Rhino-Laryngology, Head and Neck Surgery (Deutsche Gesellschaft für Hals-Nasen-Ohren-Heilkunde, Kopf- und Hals-Chirurgie) |
| DGP | German Society for Pneumology (Deutsche Gesellschat für Pneumologie) |
| DGPM | German Society for Psychosomatic Medicine (Deutsche Gesellschaft für Psychosomatische Medizin) |
| GPA | German Society for Pediatric Allergology and Environmental Medicine (Gesellschaft für Pädiatrische Allergologie und Umweltmedizin) |
| GPGE | German Society for Pediatric Gastroenterology and Nutrition (Gesellschaft für pädiatrische Gastroenterologie und Ernährung) |

*Table A. 3 List of the 36 included guidelines with the abbreviation used in the other tables*

| Abbreviation | Title | Lead Association | Citation |
| --- | --- | --- | --- |
| CPGs | | | |
| Allergy prevention | | | |
| CPS 2013 | Dietary exposures and allergy prevention in high-risk infants: A joint statement with the Canadian Society of Allergy and Clinical Immunology | CPS | (Chan and Cummings 2013) |
| DGAKI 2014 | S3-Leitlinie Allergieprävention – Update 2014 | DGAKI & DGKJ | (Schäfer et al. 2014) |
| Chan 2016 | Guidelines for allergy prevention in Hong Kong | Unclear | (Chan et al. 2016) |
| SIPPS 2016 | Prevention of food and airway allergy: consensus of the Italian Society of Preventive and Social Paediatrics, the Italian Society of Paediatric Allergy and Immunology, and Italian Society of Pediatrics | SIPPS & SIAIP | (Di Mauro et al. 2016) |
| Recto 2017 | Dietary primary prevention of allergic diseases in children: the Philippine guidelines | Unclear | (Recto et al. 2017) |
| Asthma | | | |
| GINA 2011 | Global strategy for the diagnosis and management of asthma in children 5 years and younger | GINA | (Pedersen et al. 2011) |
| SIGN 2019 | British Guideline on the management of asthma | SIGN & BTS | (Scottish Intercollegiate Guidelines Network and British Thoracic Society 2019) |
| GINA 2020 | Global Strategy for Asthma Management and Prevention | GINA | (Global Initiative for Asthma 2020) |
| Atopic Eczema | | | |
| SIGN 2011 | Management of atopic eczema in primary care. | SIGN | (Scottish Intercollegiate Guidelines Network 2011) |
| AAD 2013 | Guidelines of care for the management of atopic dermatitis: Section 1. Diagnosis and assessment of atopic dermatitis | AAD | (Eichenfield et al. 2013) |
| EDF 2018 | Consensus-based European guidelines for treatment of atopic eczema (atopic dermatitis) in adults and children: part I | EDF | (Wollenberg et al. 2018) |
| AAP 2019 | The Effects of Early Nutritional Interventions on the Development of Atopic Disease in Infants and Children: The Role of Maternal Dietary Restriction, Breastfeeding, Hydrolyzed Formulas, and Timing of Introduction of Allergenic Complementary Foods | AAP | (Greer et al. 2019) |
| CDA 2019 | Approach to the Assessment and Management of Pediatric Patients With Atopic Dermatitis: A Consensus Document. Section III: Treatment Options for Pediatric Atopic Dermatitis | CDA | (Lansang et al. 2019) |
| Rajagopalan 2019 | Guidelines on Management of Atopic Dermatitis in India: An Evidence-Based Review and an Expert Consensus | Unclear | (Rajagopalan et al. 2019) |
| Food Allergies | | | |
| NIAID 2010 | Guidelines for the diagnosis and management of food allergy in the United States: report of the NIAID-sponsored expert panel | NIAID | (Boyce et al. 2010) |
| BSACI 2010 | British Society for Allergy and Clinical Immunology guidelines for the management of egg allergy | BSACI | (Clark et al. 2010) |
| AMS 2010 | Academy of medicine, Singapore-Ministry of Health clinical practice guidelines: management of food allergy | AMS | (Lee et al. 2010) |
| EAACI 2014 | EAACI food allergy and anaphylaxis guidelines. Primary prevention of food allergy | EAACI | (Muraro et al. 2014) |
| AAAAI 2014 | Food allergy: a practice parameter update-2014 | AAAAI | (Sampson et al. 2014) |
| JSPACI 2017 | Japanese guidelines for food allergy 2017 | JSPACI | (Ebisawa et al. 2017) |
| AAAAI 2017 | International consensus guidelines for the diagnosis and management of food protein-induced enterocolitis syndrome (FPIES): Executive summary-Workgroup Report of the Adverse Reactions to Foods Committee, American Academy of Allergy, Asthma & Immunology | AAAAI | (Nowak-Węgrzyn et al. 2017) |
| BSACI 2017 | BSACI guideline for the diagnosis and management of peanut and tree nut allergy | BSACI | (Stiefel et al. 2017) |
| NIAID 2017 | Addendum guidelines for the prevention of peanut allergy in the United States: Report of the National Institute of Allergy and Infectious Diseases-sponsored expert panel | NIAID | (Togias et al. 2017) |
| FBDGs | | | |
| Nutrition | | | |
| ÖGKJ 2010 | Österreichische Beikostempfehlungen | ÖGKJ | (Österreichische Gesellschaft für Kinder- und Jugendheilkunde 2010) |
| NHMRC 2012 | Infant Feeding Guidelines | NHMRC | (National Health and Medical Research Council 2012) |
| NHMRC 2013 | Australian Dietary Guidelines | NHMRC | (National Health and Medical Research Council 2013) |
| HC 2014 | Nutrition for Healthy Term Infants: Recommendations from Six to 24 Months | Health Canada, CPS, Dietitians of Canada and BCC | (Health Canada et al. 2014) |
| NCM 2014 | Nordic Nutrition Recommendations 2012 | Nordic Council of Ministers | (Nordic Council of Ministers 2014) |
| AHS 2015 | Nutrition Guideline Healthy Infants and Young Children Introduction of Complementary Foods | AHS | (Alberta Health Services 2015) |
| HC 2015 | Nutrition for healthy term infants: Recommendations from birth to six months | Health Canada, CPS, Dietitians of Canada and BCC | (Health Canada et al. 2015) |
| NGIL 2016 | Ernährung und Bewegung von Säuglingen und stillenden Frauen | NGIL (part oft he BZfE) | (Koletzko et al. 2016) |
| Perez-Escamilla 2017 | Feeding Guidelines for Infants and Young Toddlers: A Responsive Parenting Approach | Unclear | (Pérez-Escamilla et al. 2017) |
| NGIL 2018 | Diet and Lifestyle Before and During Pregnancy – Practical Recommendations of the Germany-wide Healthy Start – Young Family Network | NGIL (part oft he BZfE) | (Koletzko et al. 2018) |
| THL 2019 | EATING TOGETHER – food recommendations for families with children | THL | (National Institute for Health and Welfare in Finland 2019) |
| SGE 2019 | Ernährung des Säuglings im ersten Lebensjahr | SGE | (Schweizerische Gesellschaft für Ernährung 2019) |
| Food Allergies | | | |
| Bürklin 2019 | Ernährungsberatung bei Kindern mit IgE-vermittelten Nahrungsmittelallergien | Unclear | (Bürklin et al. 2019) |

CPS, Canadian Paediatric Society; DGAKI, Deutsche Gesellschaft für Allergologie und klinische Immunologie; DGKJ, Deutsche Gesellschaft für Kinder- und Jugendmedizin; SIPPS, Italian Society of Preventive and Social Paediatrics; SIAIP, Italian Society of Paediatric Allergy and Immunology; GINA, Global Initiative for Asthma; SIGN, Scottish Intercollegiate Guidelines Network; BTS, British Thoracic Society; AAD, American Academy of Dermatology; EDF, European Dermatology Forum; AAP, American Academy of Pediatrics; CDA, Canadian Dermatology Association; NIAID, National Institute of Allergy and Infectious Diseases; BSACI, British Society for Allergy and Clinical Immunology; AMS, Academy of medicine, Singapore; EAACI, European Academy of Allergy and Clinical Immunology; AAAAI, American Academy of Allergy, Asthma and Immunology; JSPACI, Japanese Society of Pediatric Allergy and Clinical Immunology; ÖGKJ, Österreichische Gesellschaft für Kinder- und Jugendheilkunde; NHMRC, National Health and Medical Research Counsil; AHS, Alberta Health Services; BCC, Breastfeeding Committee for Canada; NGIL, Netzwerk Gesund ins Leben – eine Initiative von IN FORM; BZfE, Bundeszentrum für Ernährung; THL, Finnish Institute for Health and Welfare, Finland; SGE, Schweizerische Gesellschaft für Ernährung

Table A. 4 Title, characteristics, and quality appraisal of included CPGs and FBDGs

| Abbreviation | Lead  Association | Full Title | Country/ Scope | AGREE II | | | | | | |
| --- | --- | --- | --- | --- | --- | --- | --- | --- | --- | --- |
|  |  |  |  | (1) | (2) | (3) | (4) | (5) | (6) | Overall |
|  |  | CPGs & FBDGs |  | 66 % | 50 % | 42 % | 79 % | 24 % | 38 % | 63 % |
|  |  | Allergy Prevention |  |  |  |  |  |  |  |  |
| CPS 2013 | CPS | Dietary exposures and allergy prevention in high-risk infants: A joint statement with the Canadian Society of Allergy and Clinical Immunology | Canada | 72 % | 31 % | 26 % | 83 % | 17 % | 0 % | 42 % |
| DGAKI 2014 | DGAKI & DGKJ^‡^ | S3-Leitlinie Allergieprävention - Update 2014^#^ | Germany | 78 % | 58 % | 67 % | 81 % | 44 % | 96 % | 92 % |
| Chan 2016 | Unclear | Guidelines for allergy prevention in Hong Kong | Hong Kong | 42 % | 8 % | 14 % | 83 % | 10 % | 46 % | 42 % |
| SIPPS 2016 | SIPPS & SIAIP | Prevention of food and airway allergy: consensus of the Italian Society of Preventive and Social Paediatrics, the Italian Society of Paediatric Allergy and Immunology, and Italian Society of Pediatrics | Italy | 72 % | 44 % | 50 % | 92 % | 23 % | 42 % | 67 % |
| Recto 2017 | Unclear | Dietary primary prevention of allergic diseases in children: the Philippine guidelines | Philippines | 64 % | 69 % | 51 % | 89 % | 15 % | 42 % | 67 % |
|  |  | Asthma |  |  |  |  |  |  |  |  |
| GINA 2011 | GINA | Global strategy for the diagnosis and management of asthma in children 5 years and younger | International | 86 % | 31 % | 34 % | 75 % | 21 % | 4 % | 58 % |
| SIGN 2019 | SIGN & BTS | British Guideline on the management of asthma | United Kingdom | 92 % | 94 % | 81 % | 97 % | 38 % | 50 % | 92 % |
| GINA 2020 | GINA | Global Strategy for Asthma Management and Prevention | International | 78 % | 56 % | 64 % | 94 % | 90 % | 75 % | 83 % |
|  |  | Atopic Eczema |  |  |  |  |  |  |  |  |
| SIGN 2011 | SIGN | Management of atopic eczema in primary care. | United  Kingdom | 83 % | 86 % | 73 % | 94 % | 40 % | 75 % | 92 % |
| AAD 2013 | AAD | Guidelines of care for the management of atopic dermatitis: Section 1. Diagnosis and assessment of atopic dermatitis | United States | 86 % | 56 % | 64 % | 83 % | 17 % | 71 % | 83 % |
| EDF 2018 | EDF | Consensus-based European guidelines for treatment of atopic eczema (atopic dermatitis) in adults and children: part I | European | 50 % | 86 % | 57 % | 92 % | 21 % | 46 % | 83 % |
| AAP 2019 | AAP | The Effects of Early Nutritional Interventions on the Development of Atopic Disease in Infants and Children: The Role of Maternal Dietary Restriction, Breastfeeding, Hydrolyzed Formulas, and Timing of Introduction of Allergenic Complementary Foods | United States | 58 % | 42 % | 27 % | 56 % | 6 % | 71 % | 50 % |
| CDA 2019 | CDA | Approach to the Assessment and Management of Pediatric Patients With Atopic Dermatitis: A Consensus Document. Section III: Treatment Options for Pediatric Atopic Dermatitis | Canada | 64 % | 36 % | 17 % | 72 % | 27 % | 88 % | 50 % |
| Rajagopalan 2019 | Unclear | Guidelines on Management of Atopic Dermatitis in India: An Evidence-Based Review and an Expert Consensus | India | 69 % | 36% | 43 % | 83 % | 13 % | 58 % | 67 % |
|  |  | Food Allergies |  |  |  |  |  |  |  |  |
| NIADI 2010 | NIAID | Guidelines for the diagnosis and management of food allergy in the United States: report of the NIAID-sponsored expert panel | United States | 81 % | 89 % | 70 % | 83 % | 33 % | 67 % | 75 % |
| BSACI 2010 | BSACI | British Society for Allergy and Clinical Immunology guidelines for the management of egg allergy | United  Kingdom | 61 % | 44 % | 36 % | 69 % | 29 % | 25 % | 67 % |
| AMS 2010 | AMS | Academy of medicine, Singapore-Ministry of Health clinical practice guidelines: management of food allergy | Singapore | 61 % | 81 % | 35 % | 81 % | 15 % | 0 % | 50 % |
| EAACI 2014 | EAACI | EAACI food allergy and anaphylaxis guidelines. Primary prevention of food allergy | European | 67 % | 78 % | 79 % | 78 % | 63 % | 88 % | 83 % |
| AAAAI 2014 | AAAAI | Food allergy: a practice parameter update-2014 | United States | 28 % | 42 % | 53 % | 83 % | 25 % | 54 % | 75 % |
| JSPACI 2017 | JSPACI | Japanese guidelines for food allergy 2017 | Japan | 31 % | 28 % | 13 % | 78 % | 21 % | 33 % | 58 % |
| AAAAI 2017 | AAAAI | International consensus guidelines for the diagnosis and management of food protein-induced enterocolitis syndrome (FPIES): Executive summary-Workgroup Report of the Adverse Reactions to Foods Committee, American Academy of Allergy, Asthma & Immunology | International | 58 % | 56 % | 49 % | 86 % | 27 % | 54 % | 75 % |
| BSACI 2017 | BSACI | BSACI guideline for the diagnosis and management of peanut and tree nut allergy | United  Kingdom | 61 % | 39 % | 44 % | 72 % | 44 % | 42 % | 50 % |
| NIAID 2017 | NIAID | Addendum guidelines for the prevention of peanut allergy in the United States: Report of the National Institute of Allergy and Infectious Diseases-sponsored expert panel | United States | 78 % | 72 % | 52 % | 92 % | 31 % | 33 % | 83 % |
|  |  | Nutrition |  |  |  |  |  |  |  |  |
| ÖGKJ 2010 | ÖGKJ | Österreichische Beikostempfehlungen | Austria | 64 % | 39 % | 33 % | 86 % | 27 % | 0 % | 58 % |
| NHMRC 2012 | NHMRC | Infant Feeding Guidelines | Australia/ New Zealand | 72 % | 53 % | 55 % | 94 % | 8 % | 29 % | 58 % |
| NHMRC 2013 | NHMRC | Australian Dietary Guidelines | Australia/ New Zealand | 97 % | 81 % | 54 % | 50 % | 35 % | 46 % | 58 % |
| HC 2014 | Health Canada, CPS, Dietitians of Canada and BCC | Nutrition for Healthy Term Infants: Recommendations from Six to 24 Months | Canada | 47 % | 28 % | 11 % | 81 % | 10 % | 0 % | 42 % |
| NCM 2014 | Nordic Council of Ministers | Nordic Nutrition Recommendations 2012 | Scandinavia/ Iceland | 69 % | 31 % | 50 % | 61 % | 0 % | 25 % | 42 % |
| AHS 2015 | AHS | Nutrition Guideline Healthy Infants and Young Children Introduction of Complementary Foods | Canada | 78 % | 28 % | 19 % | 78 % | 8 % | 4 % | 33 % |
| HC 2015 | Health Canada, CPS, Dietitians of Canada and BCC | Nutrition for healthy term infants: Recommendations from birth to six months | Canada | 47 % | 28 % | 11 % | 67 % | 8 % | 0 % | 33 % |
| NGIL 2016 | NGIL (part of the BZfE) | Ernährung und Bewegung von Säuglingen und stillenden Frauen | Germany | 81 % | 50 % | 33 % | 67 % | 4 % | 38 % | 50 % |
| Perez-Escamilla 2017 | Unclear | Feeding Guidelines for Infants and Young Toddlers: A Responsive Parenting Approach | United States | 69 % | 56 % | 35 % | 78 % | 27 % | 4 % | 58 % |
| NGIL 2018 | NGIL  (part of the BZfE) | Diet and Lifestyle Before and During Pregnancy - Practical Recommendations of the Germany-wide Healthy Start - Young Family Network | Germany | 72 % | 56 % | 41 % | 78 % | 2 % | 42 % | 67 % |
| THL 2019 | THL | EATING TOGETHER - food recommendations for families with children | Scandinavia/ Iceland | 67 % | 53 % | 23 % | 72 % | 23 % | 29 % | 67 % |
| SGE 2019 | SGE | Ernährung des Säuglings im ersten Lebensjahr | Switzerland | 44 % | 8 % | 10 % | 50 % | 13 % | 0 % | 42 % |
|  |  | Food Allergies |  |  |  |  |  |  |  |  |
| Bürklin 2019 | Unclear | Ernährungsberatung bei Kindern mit IgE-vermittelten Nahrungsmittelallergien | Switzerland | 61 % | 47 % | 29 % | 89 % | 31 % | 4 % | 67 % |
|  |  | **CPGs only** |  | **66 %** | **55 %** | **48 %** | **82 %** | **29 %** | **50 %** | **69 %** |
|  |  | **FBDGs only** |  | **67 %** | **43 %** | **31 %** | **73 %** | **15 %** | **17 %** | **52 %** |
|  |  | **Comparison of CPGs and FBDGs** | **p-value** | **0.885** | **0.118** | **0.016** | **0.022** | **0.015** | **0.001** | **0.005** |

YoP: Year of publication

CPS, Canadian Paediatric Society; DGAKI, Deutsche Gesellschaft für Allergologie und klinische Immunologie; DGKJ, Deutsche Gesellschaft für Kinder- und Jugendmedizin; SIPPS, Italian Society of Preventive and Social Paediatrics; SIAIP, Italian Society of Paediatric Allergy and Immunology; GINA, Global Initiative for Asthma; SIGN, Scottish Intercollegiate Guidelines Network; BTS, British Thoracic Society; AAD, American Academy of Dermatology; EDF, European Dermatology Forum; AAP, American Academy of Pediatrics; CDA, Canadian Dermatology Association; NIAID, National Institute of Allergy and Infectious Diseases; BSACI, British Society for Allergy and Clinical Immunology; AMS, Academy of medicine, Singapore; EAACI, European Academy of Allergy and Clinical Immunology; AAAAI, American Academy of Allergy, Asthma and Immunology; JSPACI, Japanese Society of Pediatric Allergy and Clinical Immunology; ÖGKJ, Österreichische Gesellschaft für Kinder- und Jugendheilkunde; NHMRC, National Health and Medical Research Counsil; AHS, Alberta Health Services; BCC, Breastfeeding Committee for Canada; NGIL, Netzwerk Gesund ins Leben – eine Initiative von IN FORM; BZfE, Bundeszentrum für Ernährung; THL, Finnish Institute for Health and Welfare, Finland; SGE, Schweizerische Gesellschaft für Ernährung

† For technical reasons, the references cited in the Appendix are listed in a separate reference list below (1.1 Literature cited), and the numbers of the citations here therefore do not correspond to the citations in the manuscript.

‡ This German S3-guideline allergy prevention was not valid at the time of search, but was included in the sample because it is the most comprehensive German guideline on allergy prevention

Table A. 5 Recommendation statements on the introduction of complementary foods and allergenic foods in high-risk infants

| Recommendation statement | Recommended^†^ | | Not recommended^†^ | | No recommendation made^†^ | | SoR | LoE |
| --- | --- | --- | --- | --- | --- | --- | --- | --- |
|  | CPG (Abbr.) | FBDG (Abbr.) | CPG (Abbr.) | FBDG (Abbr.) | CPG (Abbr.) | FBDG (Abbr.) |  |  |
| Introducing solid foods at  4-6 months of age |  | NGIL 2016 |  |  |  |  |  |  |
|  | EAACI 2014^‡^ |  |  |  |  |  | + | ++ |
| Introducing common food  allergens from 6 months of age | EDF 2018^‡^ |  |  |  |  |  | ++ | ++ |
|  |  | HC 2014^‡^ |  |  |  |  |  |  |
|  |  | HC 2015^‡^ |  |  |  |  |  |  |
| Delayed introduction of potential food allergens |  |  |  | HC 2015^‡^ |  |  |  |  |
|  |  |  | EAACI 2014^‡^ |  |  |  | + | ++ |
| Introducing peanut at 4-6  months of age | NIAID 2017 |  |  |  |  |  | +++ | ++ |
| Introducing peanut from 6  months of age | NIAID 2017 |  |  |  |  |  | + | + |
|  | AAP 2019 |  |  |  |  |  |  |  |
| Early introduction of peanut |  |  |  |  | BSACI 2017 |  |  |  |
|  |  |  |  |  | GINA 2020 |  |  |  |
| Consult health care providers and approach each case on an individual basis | NIAID 2017 |  |  |  |  |  |  |  |
|  |  | HC 2014 |  |  |  |  |  |  |
|  |  | Perez-Escamilla 2017 |  |  |  |  |  |  |
| Seek guidance during weaning, toensure nutrional adequacy | AAAAI 2017 |  |  |  |  |  | +++ | ++ |

Abbr., Guideline abbreviation (see table A. 3); SoR, Strength or recommendation; LoE, Level of evidence

+ weak recommendation/ low level of evidence
++ moderate recommendation/ moderate level of evidence
+++ strong recommendation/ high level of evidence
‡ Recommendation applies to children at average and at high risk for allergies

References

Alberta Health Services (2015): Nutrition Guideline Healthy Infants and Young Children Introduction of Complementary Foods. Available online at https://www.albertahealthservices.ca//nutrition/Page8567.aspx, checked on 12/12/2022.

Boyce, Joshua A.; Assa'ad, Amal H.; Burks, Wesley; Jones, Stacie M.; Sampson, Hugh A.; Wood, Robert A. et al. (2010): Guidelines for the diagnosis and management of food allergy in the United States: report of the NIAID-sponsored expert panel. In *The Journal of allergy and clinical immunology* 126 (6 Suppl), S1-58. DOI: 10.1016/j.jaci.2010.10.007.

Bürklin, Selina; Relats, Claudia; Herzog, Regula; Stalder, Karin; Roduit, Caroline; Fischer, Isabel et al. (2019): Ernährungsberatung bei Kindern mit IgE-vermittelten Nahrungsmittelallergien. Eine Praxisleitlinie, 2019. Available online at https://www.bfh.ch/dam/jcr:34f97a5a-0065-45ba-8340-fc37e0f62abe/ERNA.pdf, checked on 1/25/2024.

Chan, Alson W. M.; Chan, June K. C.; Tam, Alfred Y. C.; Leung, T. F.; Lee, T. H. (2016): Guidelines for allergy prevention in Hong Kong. In *Hong Kong medical journal = Xianggang yi xue za zhi* 22 (3), pp. 279–285. DOI: 10.12809/hkmj154763.

Chan, Edmond S.; Cummings, Carl (2013): Dietary exposures and allergy prevention in high-risk infants. A joint statement with the Canadian Society of Allergy and Clinical Immunology. In *Paediatrics & child health* 18 (10), pp. 545–554. DOI: 10.1093/pch/18.10.545.

Clark, A. T.; Skypala, I.; Leech, S. C.; Ewan, P. W.; Dugué, P.; Brathwaite, N. et al. (2010): British Society for Allergy and Clinical Immunology guidelines for the management of egg allergy. In *Clinical and experimental allergy : journal of the British Society for Allergy and Clinical Immunology* 40 (8), pp. 1116–1129. DOI: 10.1111/j.1365-2222.2010.03557.x.

Di Mauro, Giuseppe; Bernardini, Roberto; Barberi, Salvatore; Capuano, Annalisa; Correra, Antonio; De' Angelis, Gian Luigi et al. (2016): Prevention of food and airway allergy: consensus of the Italian Society of Preventive and Social Paediatrics, the Italian Society of Paediatric Allergy and Immunology, and Italian Society of Pediatrics. In *The World Allergy Organization journal* 9, pp. 1–28. DOI: 10.1186/s40413-016-0111-6.

Ebisawa, Motohiro; Ito, Komei; Fujisawa, Takao; on behalf of Committee for Japanese Pediatric Guideline for Food Allergy, The Japanese Society of Pediatric Allergy and Clinical Immunology, The Japanese Society of Allergology (2017): Japanese guidelines for food allergy 2017. In *Allergology international : official journal of the Japanese Society of Allergology* 66 (2), pp. 248–264. DOI: 10.1016/j.alit.2017.02.001.

Eichenfield, Lawrence F.; Tom, Wynnis L.; Chamlin, Sarah L.; Feldman, Steven R.; Hanifin, Jon M.; Simpson, Eric L. et al. (2013): Guidelines of care for the management of atopic dermatitis: Section 1. Diagnosis and assessment of atopic dermatitis. In *Journal of the American Academy of Dermatology* 70 (2), pp. 338–351. DOI: 10.1016/j.jaad.2013.10.010.

Global Initiative for Asthma (2020): Global Strategy for Asthma Management and Prevention. Updated 2020. Available online at www.ginasthma.org, checked on 8/4/2020.

Greer, Frank R.; Sicherer, Scott H.; Burks, A. Wesley (2019): The Effects of Early Nutritional Interventions on the Development of Atopic Disease in Infants and Children: The Role of Maternal Dietary Restriction, Breastfeeding, Hydrolyzed Formulas, and Timing of Introduction of Allergenic Complementary Foods. In *Pediatrics*. DOI: 10.1542/peds.2019-0281.

Health Canada; Canadian Paediatric Society; Dietitians of Canada; Breastfeeding Committee for Canada (2014): Nutrition for Healthy Term Infants: Recommendations from Six to 24 Months. Available online at https://www.canada.ca/en/health-canada/services/canada-food-guide/resources/infant-feeding/nutrition-healthy-term-infants-recommendations-birth-six-months/6-24-months.html, checked on 12/12/2022.

Health Canada; Canadian Paediatric Society; Dietitians of Canada; Breastfeeding Committee for Canada (2015): Nutrition for healthy term infants: Recommendations from birth to six months. Available online at https://www.canada.ca/en/health-canada/services/canada-food-guide/resources/infant-feeding/nutrition-healthy-term-infants-recommendations-birth-six-months.html#a4, checked on 12/12/2022.

Koletzko, Berthold; Bauer, Carl-Peter; Cierpka, M.; Cremer, Monika; Flothkötter, Maria; Graf, Christine et al. (2016): Ernährung und Bewegung von Säuglingen und stillenden Frauen. Aktualisierte Handlungsempfehlungen von "Gesund ins Leben - Netzwerk Junge Familie", eine Initiative von IN FORM. In *Monatsschrift Kinderheilkunde* 164 (S5), pp. 433–457. Available online at https://www.gesund-ins-leben.de/, checked on 12/12/2022.

Koletzko, Berthold; Cremer, Monika; Flothkötter, Maria; Graf, Christine; Hauner, Hans; Hellmers, Claudia et al. (2018): Ernährung und Lebensstil vor und während der Schwangerschaft - Handlungsempfehlungen des bundesweiten Netzwerks Gesund ins Leben. In *Geburtshilfe und Frauenheilkunde* 78 (12), pp. 1262–1282. DOI: 10.1055/a-0713-1058.

Lansang, Perla; Lam, Joseph M.; Marcoux, Danielle; Prajapati, Vimal H.; Spring, Shanna; Lara-Corrales, Irene (2019): Approach to the Assessment and Management of Pediatric Patients With Atopic Dermatitis: A Consensus Document. Section III: Treatment Options for Pediatric Atopic Dermatitis. In *Journal of cutaneous medicine and surgery* 23 (5_suppl), 19S-31S. DOI: 10.1177/1203475419882647.

Lee, Bee Wah; Aw, M. M.; Chiang, W. C.; Daniel, M.; George, G. M.; Goh, E. N. et al. (2010): Academy of medicine, Singapore-Ministry of Health clinical practice guidelines: management of food allergy. In *Singapore medical journal* 51 (7), pp. 599–607. Available online at http://smj.sma.org.sg/5107/5107cpg1.pdf, checked on 12/22/2022.

Muraro, Antonella; Halken, Susanne; Arshad, Syed Hasan; Beyer, Kirsten; Dubois, Anthony E. J.; Du Toit, George et al. (2014): EAACI food allergy and anaphylaxis guidelines. Primary prevention of food allergy. In *Allergy* 69 (5), pp. 590–601. DOI: 10.1111/all.12398.

National Health and Medical Research Council (2012): Infant Feeding Guidelines. Available online at https://www.nhmrc.gov.au/, checked on 12/9/2022.

National Health and Medical Research Council (2013): Australian Dietary Guidelines. Available online at https://www.nhmrc.gov.au/, checked on 12/12/2022.

National Institute for Health and Welfare in Finland (2019): EATING TOGETHER - food recommendations for families with children. 2. Auflage. Available online at https://urn.fi/URN:ISBN:978-952-343-264-2, checked on 12/9/2022.

Nordic Council of Ministers (2014): Nordic Nutrition Recommendations 2012. Integrating nutrition and physical activity. 5. Auflage. Copenhagen. Available online at http://dx.doi.org/10.6027/Nord2014-002, checked on 12/9/2022.

Nowak-Węgrzyn, Anna; Chehade, Mirna; Groetch, Marion E.; Spergel, Jonathan M.; Wood, Robert A.; Allen, Katrina J. et al. (2017): International consensus guidelines for the diagnosis and management of food protein-induced enterocolitis syndrome: Executive summary-Workgroup Report of the Adverse Reactions to Foods Committee, American Academy of Allergy, Asthma & Immunology. In *The Journal of allergy and clinical immunology* 139 (4), 1111-1126.e4. DOI: 10.1016/j.jaci.2016.12.966.

Österreichische Gesellschaft für Kinder- und Jugendheilkunde (2010): Österreichische Beikostempfehlungen. Richtig essen von Anfang an! Available online at https://www.richtigessenvonanfangan.at/home, checked on 12/23/2022.

Pedersen, Søren; Hurd, Suzanne S.; Lemanske, Robert F., JR; Becker, Allan B.; Zar, Heather J.; Sly, Peter D. et al. (2011): Global strategy for the diagnosis and management of asthma in children 5 years and younger. In *Pediatric pulmonology* 46 (1), pp. 1–17. DOI: 10.1002/ppul.21321.

Pérez-Escamilla, Rafael; Segura-Pérez, Sofia; Lott, Megan (2017): Feeding Guidelines for Infants and Young Toddlers: A Responsive Parenting Approach. In *Nutrition Today* 52 (5), 223-231. DOI: 10.1097/NT.0000000000000234.

Rajagopalan, Murlidhar; De, Abhishek; Godse, Kiran; Krupa Shankar, D. S.; Zawar, Vijay; Sharma, Nidhi et al. (2019): Guidelines on Management of Atopic Dermatitis in India: An Evidence-Based Review and an Expert Consensus. In *Indian journal of dermatology* 64 (3), pp. 166–181. DOI: 10.4103/ijd.IJD_683_18.

Recto, Marysia Stella T.; Genuino, Maria Lourdes G.; Castor, Mary Anne R.; Casis-Hao, Roxanne J.; Tamondong-Lachica, Diana R.; Sales, Maria Imelda V. et al. (2017): Dietary primary prevention of allergic diseases in children: the Philippine guidelines. In *Asia Pacific allergy* 7 (2), pp. 102–114. DOI: 10.5415/apallergy.2017.7.2.102.

Sampson, Hugh A.; Aceves, Seema; Bock, S. Allan; James, John; Jones, Stacie M.; Lang, David et al. (2014): Food allergy: a practice parameter update-2014. In *The Journal of allergy and clinical immunology* 134 (5), 1016-25.e43. DOI: 10.1016/j.jaci.2014.05.013.

Schäfer, Torsten; Bauer, Carl-Peter; Beyer, Kirsten; Bufe, A.; Friedrichs*, F.; Gieler, Uwe et al. (2014): S3-Leitlinie Allergieprävention - Update 2014. Leitlinie der Deutschen Gesellschaft für Allergologie und klinische Immunologie (DGAKI) und der Deutschen Gesellschaft für Kinder- und Jugendmedizin (DGKJ). Available online at https://www.awmf.org, checked on 3/10/2020.

Schweizerische Gesellschaft für Ernährung (2019): Ernährung des Säuglings im ersten Lebensjahr. Available online at http://www.sge-ssn.ch/media/Merkblatt_Ernaehrung_des_Saeuglings_im_ersten_Lebensjahr-2019.pdf, checked on 12/12/2022.

Scottish Intercollegiate Guidelines Network (2011): Management of atopic eczema in primary care. A national clinical guideline. (SIGN Guideline No 125). Edinburgh (125). Available online at http://www.sign.ac.uk, checked on 7/30/2020.

Scottish Intercollegiate Guidelines Network; British Thoracic Society (2019): British Guideline on the management of asthma. A national clinical guideline. (SIGN Guideline No 158). Edinburgh (158). Available online at http://www.sign.ac.uk, checked on 7/30/2020.

Stiefel, G.; Anagnostou, Katherine; Boyle, Robert J.; Brathwaite, N.; Ewan, P. W.; Fox, Adam T. et al. (2017): BSACI guideline for the diagnosis and management of peanut and tree nut allergy. In *Clinical and experimental allergy : journal of the British Society for Allergy and Clinical Immunology* 47 (6), pp. 719–739. DOI: 10.1111/cea.12957.

Togias, Alkis; Cooper, Susan F.; Acebal, Maria L.; Assa'ad, Amal H.; Baker, James R.; Beck, Lisa A. et al. (2017): Addendum guidelines for the prevention of peanut allergy in the United States: Report of the National Institute of Allergy and Infectious Diseases-sponsored expert panel. In *The Journal of allergy and clinical immunology* 139 (1), pp. 29–44. DOI: 10.1016/j.jaci.2016.10.010.

Wollenberg, Andreas; Barbarot, Sébastien; Bieber, Thomas; Christen-Zaech, S.; Deleuran, Mette; Fink-Wagner, Antjie et al. (2018): Consensus-based European guidelines for treatment of atopic eczema (atopic dermatitis) in adults and children: part I. In *Journal of the European Academy of Dermatology and Venereology : JEADV* 32 (5), pp. 657–682. DOI: 10.1111/jdv.14891.
